# Эндокринные аспекты старения человека

**DOI:** 10.14341/probl13774

**Published:** 2026-09-08

**Authors:** Р. К. Михеев, О. Р. Григорян, Н. Н. Волеводз, Р. В. Роживанов, Е. В. Шереметьева, Е. Н. Андреева, Г. А. Мельниченко

**Affiliations:** Национальный медицинский исследовательский центр эндокринологии им. акад. И.И. Дедова Россия; Endocrinology Research Centre Russian Federation; Национальный медицинский исследовательский центр эндокринологии им. акад. И.И. Дедова; Московский областной научно-исследовательский клинический институт им. М.Ф. Владимирского Россия; Endocrinology Research Centre; Moscow Regional Research and Clinical Institute Russian Federation; Национальный медицинский исследовательский центр эндокринологии им. акад. И.И. Дедова; Российский университет медицины Россия; Endocrinology Research Centre; Russian University of Medicine Russian Federation

**Keywords:** старение, менопауза, гиперандрогения, андропауза, адренопауза, соматопауза, гипопролактинемия, ageing, menopause, hyperandrogenism, andropause, adrenopause, somatopause, hypoprolactinemia

## Abstract

Старение — многофакторный процесс, запрограммированный на генетическом и эпигенетическом уровнях, затрагивающий все органы и системы. На современном этапе старение является крайне актуальной проблемой не только в научном, но и в социально-демографическом плане. Несмотря на то, что связь биологического старения с накоплением нарушений эндокринной регуляции очевидна, проведенные за последние 150 лет исследования, в поисках потенциального эндокринного «эликсира долголетия», являлись методологически неоднородными и спорадическими, а их результаты — противоречивыми, взаимоисключающими и даже обескураживающими. Парадоксально, но в последние годы сформировалась позиция трактовать изменения эндокринной регуляции в старшем возрасте не только как угасание, но и как «адаптационная перестройка», цель которой — достижение «благополучного старения». В данном обзоре представлены современные данные по особенностям эндокринного угасания функции щитовидной железы, соматотропной (соматопауза) и пролактинпродуцирующей (гипопролактинемия) функций гипофиза, надпочечников (гиперкортицизм и адренопауза), мужской (андропауза) и женской (менопауза, гиперандрогения) половой системы.

## Abstract (en)

Ageing is multifactorial, genetically and epigenetically programmed biological process. Nowadays ageing is acknowledged as extremely actual scientifical, social and demographical problem. Despite the evidence of interactions between ageing and accumulation of endocrine disruptions, the last 150 years of variegated searches for potential endocrine “elixir of lon gevity” brought contradictive, mutually exclusive and even discouraging results. Fortunately, the discovery of referent lab oratory intervals of hormonal blood levels among elderly people improved the overall situation. Traditional treatment of endocrine hormonal deficiency with replacement therapy does not lead to rejuvenation but to potential damage. That’s why it is more accepted to interpret hormonal changes among elderly people not only as aging but specific adaptation for “successful ageing”. The present review contains modern datas about endocrine ageing of thyroid gland, adrenal glands (hy percorticism, adrenopause), pituitary gland (somatopause, hypoprolactinemia), male (andropause) and female (menopause, hyperandrogenism) hypogonadism.

## ВВЕДЕНИЕ

Освоение клинической специальности «эндокринология» подразумевает овладение навыком правильной интерпретации результатов лабораторных и биохимических анализов органов эндокринной системы в комплексе с топическими данными и клинической картиной. Одной из наиболее распространенных врачебных ошибок является предвзятая оценка уровня гормональных маркёров без учета возрастных особенностей, что опасно с точки зрения снижения качества и продолжительности жизни у пожилых людей в мире. Согласно информационному бюллетеню ВОЗ от 01.10.2025 г., численность населения Земли в возрасте 60 лет и старше к 2050 г. удвоится и составит в итоге 2,1 млрд человек. Ожидается, что за период с 2020 по 2050 г. численность населения в возрасте 80 лет и старше вырастет практически втрое и достигнет 426 млн человек [1][2].

Функционирование желез внутренней секреции в периоде угасания имеет свои отличительные черты и особенности у каждого отдельно взятого индивидуума старшего возраста. Общими патогенетическими механизмами, лежащими в основе развития данного процесса, являются [2]: 1) прогрессирующее снижение числа гормонпродуцирующих клеток (пример: снижение выработки тестостерона за счет постепенного снижения числа клеток Лейдига у мужчин); 2) аномалии циркадных ритмов и увеличение/уменьшение суточной амплитуды секреции гормонов (увеличение суточной секреции кортизола за счет вечерней фракции); 3) аномалии сигналинга в результате повреждения рецепторов и нарушения работы посредников (нарушения в PI3K/AKT/mTOR, инсулин/ИФР-1 сигнальном пути) [3][4]. Так, например, возраст-обусловленное снижение секреции ГР (гормона роста) клиницист может понимать под разными ракурсами: с одной стороны — как предиктор ожирения, сахарного диабета 2 типа (СД2) и саркопении [5] и показанием к инициации терапии гормоном роста, с другой — как своеобразный «иммунитет» от потенциальных злокачественных новообразований [6].

## ЩИТОВИДНАЯ ЖЕЛЕЗА

По данным исследований, опубликованных Monzani et al. (1996) [7], циркадные ритмы секреции ТТГ отличаются стабильностью вплоть до глубокой старости. В 1980-е и 1990-е годы были инициированы попытки экспериментально обнаружить лабораторные предикторы «старения» щитовидной железы. В 1991 г. по результатам оценки уровня ТТГ в рамках пробы с синтетически аналогом тиреотропин-рилизинг гормона между группами с 11 (2 мужчин и 9 женщин) здоровыми пациентами пожилого (81–94 лет) возраста и 11 (2 мужчин и 9 женщин) пациентов аналогичного возраста с болезнью Альцгеймера [8] не было выявлено каких-либо статистически значимых различий на 20 (р=0,71), 30 (р=0,26), 45 минутах (р=0,28) исследования. В период с 1988 по 1994 гг. по итогам 3 этапа Национального обследования состояния здоровья и питания (National Health and Nutrition Examination Survey, NHANES III) [9] в США было обнаружено, что у лиц с отсутствием заболеваний щитовидной железы и выработки аутоантител в анамнезе, достаточным потреблением йода и приемом потенциальных фармакологических дизрапторов уровень ТТГ имеет тенденцию к прогрессивному росту, параллельно возрасту, соответственно лабораторной картине субклинического гипотиреоза на фоне незначительного роста уровня свТ4 и снижения уровня свТ3. Вариабельность медианных и 97,5-перцентильных значений ТТГ составила в группе участников 20–29 лет от 1,26 до 3,56 мЕд/л, в группе 80+ лет — от 1,90 до 7,49 мЕд/л. Результирующее действие комбинации снижения чувствительности тиреотрофов гипофиза к периферическим гормонам щитовидной железы, снижение чувствительности щитовидной железы к низкоактивному ТТГ, а также снижение дейодирования Т4 в Т3 создает у клиницистов соблазн к повальному назначению заместительной терапии левотироксином натрия пожилым пациентам. Такая тактика опасна с точки зрения повышения сердечно-сосудистых рисков у потенциально скомпрометированных в отношении хронических заболеваний пациентов и обуславливает необходимость принятия для пожилых пациентов собственных референсных лабораторных интервалов ТТГ. Согласно клиническим рекомендациям американской тиреоидологической ассоциации от 2014 г. (ATA, American Thyroid Association) назначение такого рода терапии пожилым пациентам должно проводиться строго с учетом рисков полипрагмазии, коморбидности, «клинических масок» проявлений гипотиреоза и при условии титрования с минимально возможной дозой начальной дозой из расчета 0,3–0,4 мкг/кг/сут [10].

## ГИПЕРКОРТИЦИЗМ

Одним из потенциальных эндокринных геростимулирующих факторов является активация гипоталамо-гипофизарно-надпочечниковой оси. В основе данного процесса лежит более раннее время достижения пика секреции кортизола, а также повышение вечернего кортизола со смещением на 1–2 часа назад по сравнению с пациентами молодого возраста [11]. По данным метаанализа 45 кросс-секционных исследований (n=1200) у пациентов пожилого возраста отмечалась более высокая выработка кортизола в ответ на физиологическую и фармакологическую стимуляцию (стрессы, тест с инсулиновой гипогликемией, введение препаратов кортиколиберина, адренокортикотропного гормона, антагонистов опиодных рецепторов), а также на подавляющие тесты с дексаметазоном и преднизолоном [12]. Кроме того, по данным Ennis GE et al. (2017), повышение уровня суточной кортизолурии тесно ассоциировалось с риском развития болезни Альцгеймера [13]. По результатам многолетнего (2002–2019 гг.) исследования с применением ПЭТ с флортауципиром (¹⁸F) и Питтсбургским соединением B (¹¹C) у постменопаузальных пациенток была обнаружена прямая зависимость между уровнем кортизола в сыворотке в 08:00 утра и выраженностью отложения бета- и тау-амилоида в различных зонах коры головного мозга (по Z-критерию): коре задней части поясной извилины (0,116 SD±0,038, p=0,003), коре углубления верхней височной борозды (0,050 SD±0,022, p=0,024), лобно-латерально-ретроспленальной коре (0,075 SD±0,020, p≤0,001) головного мозга [14]. В то же время более низкая суточная секреция кортизола в пожилом возрасте ассоциировалась с большей продолжительностью сна, более низким риском развития саркопении и СД2 [15].

## АДРЕНОПАУЗА

Считается, что характер секреции таких надпочечниковых андрогенов, как ДГЭА и ДГЭА-С неоднократно меняется в периоде онтогенеза. После рождения человека уровень ДГЭА и ДГЭА-С, стремительно падает в первые дни жизни и остается сниженным примерно до 6–8 лет жизни, когда за счет созревания сетчатой зоны коры надпочечников (у девочек, как правило, раньше, чем у мальчиков) проявляются такие предваряющие половое созревание симптомы, как: лобковое и подмышечное оволосение, повышение секреции потовых и сальных желёз. Несмотря на то, что в подростковом возрасте у мальчиков основная доля андрогенной секреции приходится на клетки Лейдига, как у мальчиков, так и у девочек, уровень ДГЭА и ДГЭА-С медленно достигает своего пика к третьей декаде жизни, с последующим прогрессивным снижением на 3–5% в год [16]. В итоге, к 50 годам жизни уровень секреции надпочечниковых андрогенов достигает 40%, к восьмой-девятой декаде жизни — 10–20% от пикового уровня секреции. Несмотря на то, что секреция кортизола не подчинена к такого рода закономерности, данный процесс по аналогии с менопаузой у женщин и андропаузой у мужчин получил название «адренопауза». В конце 1990-х гг. были выдвинуты теории, рассматривающие в качестве триггеров снижения выработки ДГЭА и ДГЭА-С снижение активности 17,20-лиазы [17], атрофию сетчатой зоны коры надпочечников [18], снижение ИФР-1 и ИФР-3 [19][22].

Благодаря результатам мультисекционного многолетнего эпидемиологического американского «Исследования здоровья женщин по всей стране» (Study of Women’s Health Across the Nation, SWAN)» в последние годы намечается тенденция к пересмотру ранее сложившихся взглядов на адренопаузу [20]. Было выявлено, что у большинства женщин (85% исследованных) несмотря на общее возраст-зависимое снижение выработки андрогенов, выработка надпочечниковой фракций ДГЭА-С андрогенов росла в течение небольшого периода от ранней перименопаузы до ранней постменопаузы независимо от продукции эстрогенов яичниками, что подтвердилось у женщин как с хирургической менопаузой, так и с интактными яичниками [21].

Физиологическая роль ДГЭА и ДГЭА-С как прекурсоров синтеза андрогенов и эстрогенов вкупе с протективными эффектами в отношении рисков сахарного диабета 2 типа, остеопороза и ожирения у лабораторных животных сформировали предубеждение о данных гормонах, как многообещающих «эликсиров бессмертия» Homo Sapiens [23]. Одной из основных мишеней ДГЭА является ядерный рецептор эстрогена β (ERβ). Следующей по силе воздействия мишенью ДГЭА является рецептор андрогена (AR). ДГЭА обладает сродством к AR, которое в два-три раза ниже, чем к ERβ. ДГЭА связывается с ERα с константой равновесного связывания (Kis), равной 1,1 мкМ, и с ERβ с Kis, равной 0,5 мкМ, с преимущественным агонистическим действием на ERβ и EC50, равным примерно 200 нМ. Благодаря внутрикринной регуляции ДГЭА способен оказывать уникальное воздействие, а именно повышать уровень андрогенов, если он снижен до нормы и не превышает необходимого уровня [23]. Количество обзоров относительно терапии прогормоном возраст-ассоциированных патологий невелико, однозначных выводов нет [46][47]. Неизвестно, улучшают ли добавки с ДГА когнитивные способности у пожилых женщин. Из 15 статей, отобранных для полнотекстового анализа, четыре соответствовали критериям включения. Во всех исследованиях ДГА вводился в виде пероральной суточной дозы 50 мг, и все они были двойными слепыми. Улучшение когнитивных функций при лечении ДГА по сравнению с группой плацебо не наблюдалось ни в одном другом исследовании [48].

На данный момент применение ДГЭА как лекарственного средства разрешено вагинально. В качестве интравагинального средства прастерон был зарегистрирован FDA (2016), EMA (2017) и Министерства здравоохранения Канады (2019). Клинические испытания подтвердили эффективность ДГЭА при ежедневном интравагинальном введении 0,50% (6,5 мг) для лечения вульвовагинальной атрофии и диспареунии. Более того, действие ДГЭА не распространяется на эндометрий; он действует только во влагалище, а уровень половых стероидов в сыворотке крови остается в пределах биологически низкого диапазона нормы для постменопаузы, что исключает стимуляцию и без того атрофированного эндометрия. Механизмы положительного воздействия ДГЭА на атрофию слизистой оболочки до сих пор неизвестны [24][25].

## АНДРОПАУЗА И ГИПОПРОЛАКТИНЕМИЯ

Возраст-обусловленное снижение активности гипоталамо-гипофизарно-гонадной оси у мужчин происходит с постепенно увеличивающейся скоростью: в период 24–26 лет — 0,85%/год, с 36 до 70 лет — 1,6%/год, после 70 лет — 1,6%/год [2]. Основополагающими механизмами данного процесса является снижение числа активных клеток Лейдига в яичках (оксидативный стресс, нарушение транспорта холестерина в митохондриях), снижение пульсаторного выброса ЛГ и ФСГ по механизму отрицательной обратной связи и усиление периферической конверсии андрогенов в эстрогены в жировой ткани вследствие наличия коморбидности (ожирение, АГ, инсулинорезистентность, СД2) [26].

В современной научной литературе, к сожалению, отмечается очень много противоречий в терминологическом описании дефицита тестостерона у мужчин. Термин «мужской гипогонадизм» используется в руководствах по эндокринологии Эндокринного общества (ES) и Австралийского эндокринного общества (AUS) [49–51]. При этом в руководствах по урологии и сексуальной медицине таких ассоциаций, как Международное общество сексуальной медицины (ISSM), Британское общество сексуальной медицины (BSSM), Американская урологическая ассоциация (AUA), как правило, употребляется понятие «синдром дефицита тестостерона», который по сути является синонимом мужского гипогонадизма [28][49][52–54]. В МКБ10 синонимом гипогонадизма является диагноз E29.1 — Гипофункция яичек. При этом термины «ADAM» — androgen deficiency adult men, «PADAM» — partial androgen deficiency adult men, «мужской климакс» и «андропауза» являются устаревшими, и по сути неверными, так как не отражают патогенез гипогонадизма, а последние два термина вообще противоречат биологической сути явления. Еще более старым термином является евнухоидизм, который частично отражает биологическую суть явления в случаях развития гипогонадизма до или во время полового развития, но из-за негативного восприятия пациентами в современной медицине этот термин не используется. Наиболее верное определение мужского гипогонадизма это: клинический и биохимический синдром, связанный с низким уровнем тестостерона, обусловленным снижением эндокринной функции яичка. Таким образом, наиболее оптимально считать гипогонадизмом только ситуации, при которых уровень тестостерона снижается ниже нормальных значений (по современным данным — ниже 12 нмоль/л) вследствие повреждения центральных или периферических звеньев гипоталамо-гипофизарно-гонадной оси (ГГГО) или их сочетания. Если нарушена функция яичек без нарушения гипоталамо-гипофизарной функции, то такой гипогонадизм является первичным и гипергонадотропным (избыточная стимулирующая секреция гонадотропинов нормально функционирующим гипофизом в ответ на снижение продукции тестостерона яичком). Если нарушена гипоталамо-гипофизарная функция без нарушения функции яичек, то такой гипогонадизм является гипогонадотропным и вторичным или третичным в зависимости от исходного поражения гипофиза или гипоталамуса соответственно (снижение продукции тестостерона яичком как результат снижения стимулирующего влияния гипофиза). Если нарушены как гипоталамо-гипофизарная функция, так и функция яичек, то такой гипогонадизм является смешанным. Наиболее типичным примером смешанного гипогонадизма является тот тип гипогонадизма, который развивается по мере старения мужчины, его также называют «возрастной андрогенный дефицит», что менее точно, или «возрастной гипогонадизм», что является верным. И наконец, четвертым вариантом гипогонадизма является тот, который связан с нарушениями механизмов отрицательной обратной связи в системе «гипоталамус-гипофиз-гонады» и развивающийся на фоне метаболических нарушений, таких как ожирение и нарушение углеводного обмена. Это предполагает функциональный характер гипогонадизма и его потенциальную обратимость при устранении сопутствующих негативных факторов в отличие от первичного (гипергонадотропного), вторичного (гипогонадотропного) или смешанного типов гипогонадизма, которые являются органическими и необратимыми [55–56]. Разделение гипогонадизма на органический и функциональный имеет практический смысл: органический гипогонадизм требует лечения с помощью андрогенной терапии, а функциональный гипогонадизм должен лечиться в первую очередь путем устранения или улучшения ассоциированных с ним метаболических нарушений [57–58].

Различные работы последних десятилетий описывают наличие у участников гипотетического дважды подтвержденного лабораторного феномена, клинические проявления которого остаются до сих пор неизученными — гипопролактинемии. С целью увеличения достоверности анализов из числа участников исключались пациенты с заболеваниями гипоталамо-гипофизарной области в анамнезе, уровнем общего пролактина сыворотки ≥35 нг/мл (700 мЕд/л), приемом антипсихотиков, селективных ингибиторов обратного захвата серотонина, агонистов дофамина. Математически рассчитанная вероятность СД2 у мужчин с уровнем общего пролактина сыворотки в пределах референсного интервала 5–34,9 нг/мл (100–698 мЕд/л) составляла 6,3%, при уровне сывороточного пролактина в промежутке 3–4,9 нг/мл (60–98 мЕд/л) — 9,3%, ОР (95% ДИ 1,59 (0,93–2,71), в промежутке 0,3–2,9 нг/мл (6–58 мЕд/л) — 22,7%, OР 5,45 (95% ДИ 1,78–16,62). При этом лабораторная гипопролактинемия не ассоциировалась с повышением риска развития онкологических, сердечно-сосудистых заболеваний, депрессивных расстройств и общей смертности. Таким образом, в исследовании EMAS в качестве условной «отрезной точки» клинически значимого риска развития СД2 у мужчин европейской популяции было предложено считать уровень сывороточного пролактина 3 нг/мл и ниже (64 мЕд/л и ниже) [29].

## ПЕРИОД МЕНОПАУЗАЛЬНОГО ПЕРЕХОДА И ФИЗИОЛОГИЧЕСКИЙ СДВИГ В СТОРОНУ ОТНОСИТЕЛЬНОГО ПРЕОБЛАДАНИЯ АНДРОГЕНОВ

Репродуктивный период у женщины сменяется периодом менопаузального перехода (т.н. перименопаузальным периодом), за которым следуют менопауза и, далее — постменопауза (ранняя и поздняя) согласно системе STRAW+10. Термин «менопауза» (в пер. с лат. “mens” — месячные и “pausa” — прекращение, остановка) зачастую трактуется лишь как эстрогендефицит-обусловленное прекращение менструаций и утрата репродуктивной функции у женщины. Постменопауза характеризуется «выключением» функции яичников и развитием дефицита эстрогенов. В среднем снижается уровень эстрогенов после наступления менопаузы на 67%, Последующее снижение уровня эстрогенов, наступает через 6–8 лет после наступления менопаузы (еще на 18%) [31]. Снижение основного женского стероида ассоциировано с повышением риска развития возраст-ассоциированных заболеваний: ишемической болезни сердца (ИБС), цереброваскулярной болезни (ЦВБ), СД2, остеопороза и т.д. [31].

В результате, ввиду недостаточности половых женских гормонов, по механизму обратной связи формируется лабораторная картина гипергонадотропного гипогонадизма [32]. Ранний менопаузальный переход — это регулярный цикл с наличием стабильных колебаний его продолжительности (≥7 дней), в сочетании со следующими особенностями:

Поздний менопаузальный переход — это уже более грубые нарушения менструального цикла (аменорея ≥60 дней), а также:

Период раннего менопаузального перехода характеризуется увеличением частоты ановуляторных циклов, атрезией фолликулов или их персистенцией, формированием неполноценного желтого тела, что приводит к постепенному снижению концентрации прогестерона [40]. В период раннего менопаузального перехода наблюдаются колебания уровня ФСГ. Более высокие уровни ФСГ инициируют фолликулогенез до регрессии желтого тела, что приводит к укорочению фолликулярной фазы. Поскольку количество фолликулов продолжает уменьшаться, может наступить задержка, прежде чем появится следующий реагирующий фолликул. Даже при наличии задержек фолликул выделяет высокий уровень эстрадиола. Несмотря на нарушения фолликулогенеза, колебания уровня эстрадиола становятся все более выраженными [41–44] (рис. 1).

**Figure:**
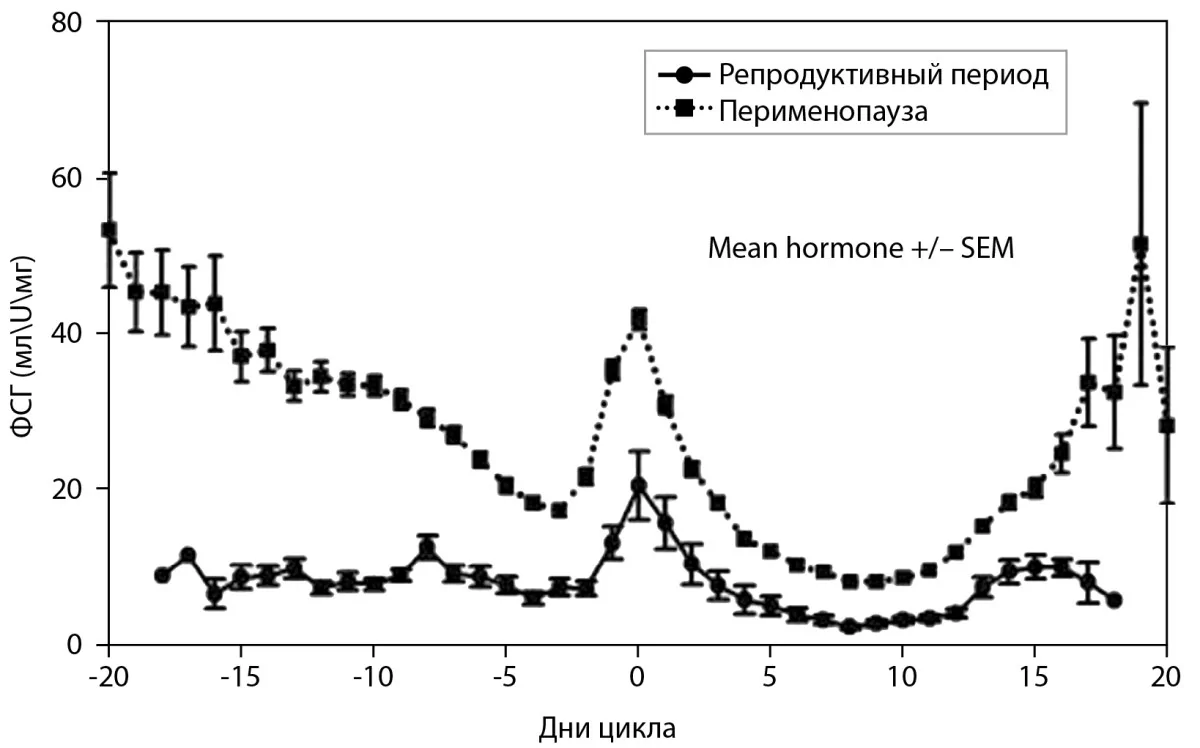
Рисунок 1. Уровни ФСГ в различные периоды жизни женщины.

В исследованиях SWAN, Penn Ovarian Aging Study и других независимо показано, что средний уровень эстрадиола у женщин в ранней перименопаузе аналогичен уровню у женщин репродуктивного возраста или выше. Эти колебания уровней эстрогенов описываются как «гормональные американские горки» или «гормональный хаос» [41–44].

Постменопауза не характеризуется окончательным затуханием продукции эстрогенов в организме женщин. В данном периоде синтез эстрогенов продолжается, но в значительно более низких концентрациях, чем в репродуктивном периоде, и с другим соотношением фракций. Синтез эстрогенов происходит в основном за счет превращения андростендиона в эстрон (в большей степени; уровень продукции в среднем 42 мкг/сутки, циркулирующий уровень в сыворотке 35 пг/мл) и тестостерона в эстрадиол (уровень циркулирующего эстрадиола в среднем <20 пг/мл) под воздействием ароматазы. В отличие от быстрого снижения уровней эстрадиола и прогестерона, уровень циркулирующего тестостерона снижается более плавно, вследствие постепенного снижения секреции как надпочечниками, так и яичниками. Концентрация тестостерона в сыворотке крови достигает пика в конце периода полового созревания, после чего сохраняется на стабильном уровне вплоть до 80-летнего возраста. При этом уровень тестостерона остается в пределах референсных значений (рис. 2) [45].

**Figure:**
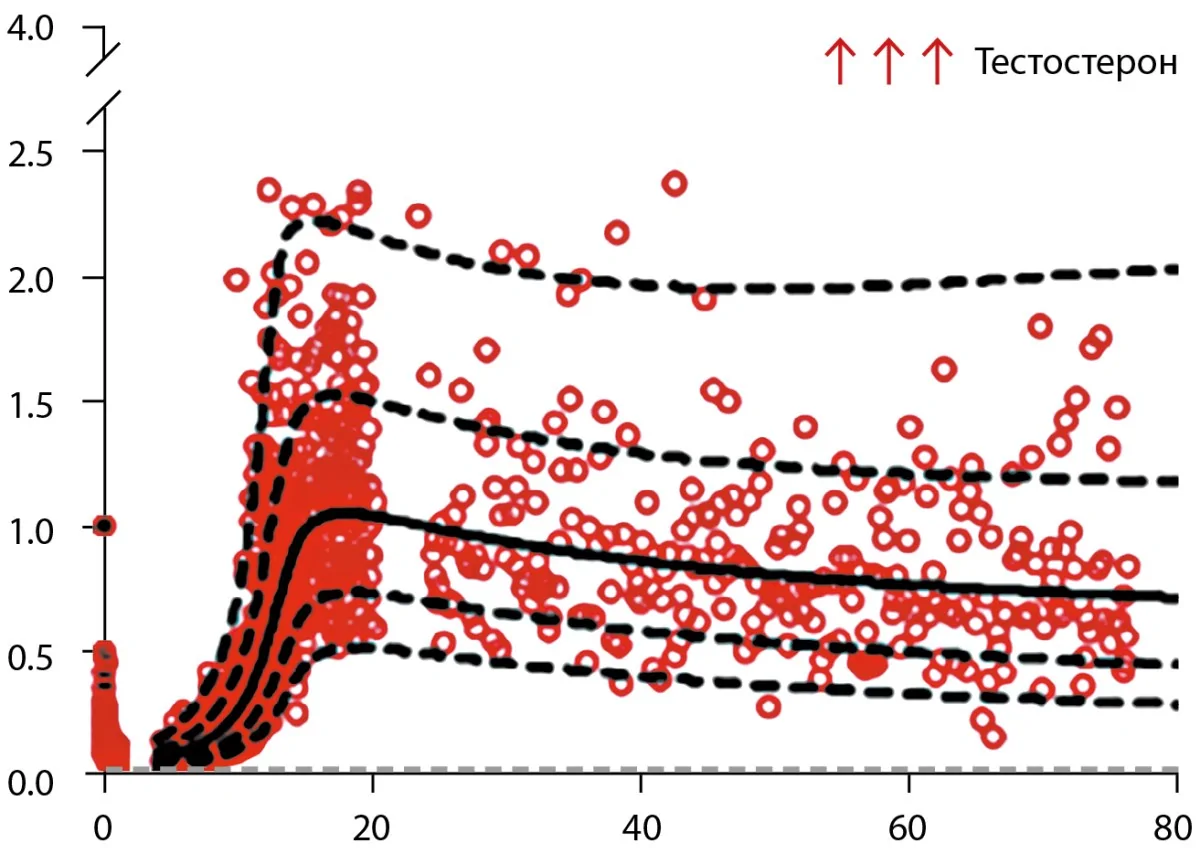
Рисунок 2. Уровень тестостерона в зависимости от возраста женщины. Концентрация стероидных метаболитов в сыворотке крови женщин в зависимости от возраста от 0,02 до 76,3 лет, n=1176. Сплошными линиями обозначены медианы, а пунктирными — 2,5, 16, 84 и 97,5 процентиля (что соответствует ±1 и ±2 стандартным отклонениям (SD) соответственно). Серые пунктирные линии показывают пределы обнаружения. Значения, превышающие +2 SD, обозначены знаком ↑. Уровень прогестерона в сыворотке крови 96 женщин в возрасте 13 лет был измерен в диапазоне от 2,7 до 80 нмоль/л. Анализ на стероидные гормоны продолжался 52 года. Концентрации, которые не поддавались определению или были ниже LOD, были заменены на соответствующие значения LODs/2. **Расчетное свободное время T (FT), n=1119 [45].

Менопаузальная гормонотерапия (МГТ) позволяет эффективно купировать симптомы климактерического синдрома, а также отсрочить развитие отдаленных последствий дефицита эстрогенов. При выборе терапии соблюдаются принципы современной концепции персонализации менопаузальной гормональной терапии. Начало системной МГТ необходимо рассматривать у женщин в возрасте менее 60 лет и с длительностью постменопаузы менее 10 лет. Оптимальное время для старта МГТ — период пери- и ранней постменопаузы. Отсутствуют возрастные ограничения при назначении локальной терапии эстрогенами (эстриолом) симптомов ГУМС. Терапевтическая цель заключается в использовании наиболее подходящей минимальной эффективной дозы МГТ в соответствии с целями лечения. Индивидуализация МГТ (выбор дозы, лекарственной формы препарата, его состава, режима использования) проводится с учетом возраста пациентки, стадии репродуктивного старения, гинекологических заболеваний, коморбидных состояний, предпочтений женщины [33].

В 2025 г. была опубликована Объединенная позиция российских экспертов по вопросу клинического значения гиперандрогении у женщин в ранней постменопаузе [34], которая подчеркнула, что:

1) на фоне дефицита прогестерона в период менопаузального перехода и быстрых темпов снижения уровней эстрогенов в период ранней постменопаузы, при продолжающейся выработке андрогенов в яичниках и надпочечниках происходит формирование физиологического сдвига в сторону относительного преобладания андрогенов в ранней постменопаузе. При этом абсолютные значения тестостерона соответствуют референсным для стадии репродуктивного старения;

2) физиологический сдвиг в сторону относительного преобладания андрогенов в постменопаузе ассоциирован с увеличением последующего риска развития метаболических и сердечно-сосудистых заболеваний;

3) необходимо учитывать факт формирования физиологического сдвига в сторону относительного преобладания андрогенов в ранней постменопаузе при определении тактики ведения пациенток. Наличие в арсенале практикующих врачей стратегии, позволяющей положительно влиять на звенья патогенеза метаболических и сердечно-сосудистых заболеваний, может оказать существенное положительное влияние на состояние здоровья женщин в постменопаузе.

С 1992 г. по настоящее время проводится крупное международное эпидемиологическое исследование World Health Initiative (WHI), долгосрочными задачами которого являются изучение потенциального влияния МГТ на риски соматической заболеваемости и на ожидаемую продолжительность жизни. Изначально в исследование были включены 161 808 женщин в возрасте 50–79 лет различной расовой (европеоиды, афроамериканки, азиатки, океаноиды) принадлежности с различным уровнем образования и доходов. Согласно последнему ежегодному отчету от 15 февраля 2025 г. в рамках исследования продолжают наблюдаться 42 630 пациенток в возрасте 75+ лет, из которых: в возрасте 75–79 лет — 1566 чел. (3,7%); 80–84 лет — 13 064 чел. (30,6%); 85–89 лет 14 780 чел. (34,7%); 90–94 лет — 9098 чел. (21,3%); 95–108 лет — 4122 чел. (9,7%). Полученные результаты являются многообещающими с точки зрения поисков геропротективных механизмов влияния МГТ и дальнейшего продвижения данного вида терапии для более широких выборок пациенток [35].

## СОМАТОПАУЗА

В связи с возрастным ослаблением пульсационных выбросов и снижения суточной амплитуды выработки СТГ у пожилых пациентов наблюдается медленное снижение выработки ИФР-1. Общеизвестно, что ось СТГ/ИФР-1 отвечает за поддержание процессов репарации и пролиферации клеток (особенно хрящевой и костной ткани), антиоксидантной и митохондриальной защиты пластичности головного мозга и сохранению когнитивных функций [36][37]. В то же время по результатам изучения 440 000 участников было установлено, что как сниженный, так и повышенный уровень ИФР-1 (по сравнению с возрастными нормами) ассоциирован с повышением риска сердечно-сосудистой смертности, деменции, СД2, остеопоротических событий и смертности от онкологических заболеваний по типу U-образной кривой [38]. У пациентов в когорте 63–74 лет при понижении уровня ИФР-1 по сравнению с пациентами группы 37–50 лет с аналогичной лабораторной картиной риск смертности, деменции, СД2 и остеопороза был на 20, 50, 10, 6% соответственно. В то же время повышенный уровень ИФР-1 у пациентов в когорте 63–74 лет приводил к повышению риска развития деменции, сердечно-сосудистых заболеваний и СД2 на 25, 18, 10% соответственно [2][38].

Предпринятые в 1990 г. попытки назначения препаратов гормона роста в дозе 30 мг/кг 3 раза в неделю подкожно у мужчин в возрасте 61–81 лет с уровнем ИФР-1 в нижней трети референсного возрастного диапазона способствовало в первые месяцы терапии помимо увеличения уровня ИФР-1, увеличению тощей массы тела в среднем на 8,8%, увеличению МПК в поясничном отделе позвоночника на 1,6% с последующим снижением до исходных значений 6 месяцев [39].

В связи с отсутствием однозначных данных за влияние активности соматотропной оси на течение старения, наличием широкого спектра побочных эффектов (карпальный туннельный синдром, гинекомастия, гипергликемия, отеки) от терапии препаратами гормона роста, а также отсутствие зарегистрированных в РФ препаратов ИФР-1 для проведения безопасной геропротективной терапии препаратами гормона роста, ИФР-1 и их комбинацией не представляется возможным.

## ЗАКЛЮЧЕНИЕ

На современном этапе и в ближайшей перспективе задача по гормональному управлению старением остается малоосуществимой. Важным шагом к решению данной задачи могло бы стать определение лабораторных референсных интервалов в различных «подкогортах» пациентов старшего возраста (61–70, 71–80, 81–90, 91–100, 100+ лет) на основании результатов рационально организованных мультикогортных международных эпидемиологических исследований с созданием и поддержкой геронтологических регистров в масштабах народонаселения.

## ДОПОЛНИТЕЛЬНАЯ ИНФОРМАЦИЯ

Источники финансирования. Исследование проводится в рамках государственного задания: «Изучение фундаментальных механизмов овариальной гиперандрогении, оптимизация комплексной диагностики и персонализация лечения для снижения кардиометаболических рисков и активного долголетия», регистрационный номер 126022417901-3.

Конфликт интересов. Авторы декларируют отсутствие явных и потенциальных конфликтов интересов, связанных с содержанием настоящей статьи.

Участие авторов. Все авторы одобрили финальную версию статьи перед публикацией, выразили согласие нести ответственность за все аспекты работы, подразумевающую надлежащее изучение и решение вопросов, связанных с точностью или добросовестностью любой части работы.
